# Genetic predisposition for femoral neck stress fractures in military conscripts

**DOI:** 10.1186/1471-2156-11-95

**Published:** 2010-10-21

**Authors:** Johanna Korvala, Heini Hartikka, Harri Pihlajamäki, Svetlana Solovieva, Juha-Petri Ruohola, Timo Sahi, Sandra Barral, Jürg Ott, Leena Ala-Kokko, Minna Männikkö

**Affiliations:** 1 Oulu Center for Cell-Matrix Research, Biocenter and Department of Medical Biochemistry and Molecular Biology, University of Oulu, Oulu, Finland; 2 Department of Orthopaedic Surgery, National Military Hospital, Helsinki, Finland; 3 Centre for Military Medicine, Research Department, Helsinki, Finland; 4 Centre of Expertise for Health and Work Ability, Finnish Institute of Occupational Health, Helsinki, Finland; 5 Department of Public Health, University of Helsinki, Helsinki, Finland; 6 Gertrude H. Sergievsky Center, College for Physicians and Surgeons, Columbia University, New York, USA; 7 Beijing Institute of Genomics, Chinese Academy of Sciences, Beijing, China; 8 Connective Tissue Gene Tests, Allentown, PA, USA

## Abstract

**Background:**

Stress fractures are a significant problem among athletes and soldiers and may result in devastating complications or even permanent handicap. Genetic factors may increase the risk, but no major susceptibility genes have been identified. The purpose of this study was to search for possible genetic factors predisposing military conscripts to femoral neck stress fractures.

**Results:**

Eight genes involved in bone metabolism or pathology (*COL1A1*, *COL1A2*, *OPG*, *ESR1, VDR*, *CTR*, *LRP5*, *IL-6*) were examined in 72 military conscripts with a femoral neck stress fracture and 120 controls. The risk of femoral neck stress fracture was significantly higher in subjects with low weight and body mass index (BMI). An interaction between the *CTR *(rs1801197) minor allele C and the *VDR *C-A haplotype was observed, and subjects lacking the C allele in *CTR *and/or the C-A haplotype in *VDR *had a 3-fold higher risk of stress fracture than subjects carrying both (OR = 3.22, 95% CI 1.38-7.49, p = 0.007). In addition, the *LRP5 *haplotype A-G-G-C alone and in combination with the *VDR *haplotype C-A was associated with stress fractures through reduced body weight and BMI.

**Conclusions:**

Our findings suggest that genetic factors play a role in the development of stress fractures in individuals subjected to heavy exercise and mechanical loading. The present results can be applied to the design of future studies that will further elucidate the genetics of stress fractures.

## Background

Stress fractures are common and potentially serious exertion injuries, especially among athletes and military conscripts [1,2]. Repetitive activities such as running and marching are among the most frequently reported causes, and the approximate incidence of stress fractures in military conscripts ranges from 0.9 to 12.3% [3,4]. Stress fractures occur most commonly in the lower extremities e.g. in the tibia, metatarsals, femur or pelvis, and they manifest as localised pain that increases during exercise.

Femoral neck fractures are one of the most serious high-risk stress fractures, because displaced fatigue fractures of the femoral neck lead to long-term morbidity in a high percentage of patients [5,6]. If the fracture is detected quickly and no dislocation has occurred, the recovery rate is usually good, but displaced femoral neck stress fractures can result in devastating complications or even permanent handicap [5,7].

The pathophysiology of stress fractures is thought to be related to cyclic mechanical loading of the bone, which stimulates an incomplete remodeling response [8]. Bone is continuously degraded and renewed, and inadequate adaptation to mechanical change leads to an imbalance between microdamage and remodeling, and gradually to a fracture. There are numerous risk factors for stress fractures; a Finnish study of male military conscripts reported that tall stature, poor physical fitness, and decreased bone mineral content and bone mineral density (BMD) are factors associated with a greater risk [9].

Several observations suggest that genetic factors contribute to stress fracture susceptibility. Singer and co-workers described multiple identical stress fractures at the same anatomic sites in monozygotic twins after the sixth week of basic training in the army [10], and multiple lower limb stress fractures in the same individual have also been reported [11]. The occurrence of many stress fractures may also indicate a defective overall bone composition due to genetic factors [12]. Findings in twins and their families suggest that differences in such traits as bone size, shape, and BMD between individuals are largely attributable to genetic differences and not to environmental effects [13].

Mutations or allelic variants in the genes leading to a variety of bone pathologies that increase bone fragility, such as collagen I, (*COL1A1 *and *COL1A2*) [14,15], vitamin D receptor (*VDR*) [16], osteoprotegerin (*OPG*) [17], calcitonin receptor (*CTR*) [18], estrogen receptor (*ESR*) [19], low density lipoprotein receptor-related protein 5 (*LRP5*) [20], and interleukin 6 (*IL-6*) [21] may also increase the risk of stress fractures. Sequence variations in these genes are associated with a low peak bone mass, osteoporosis, osteogenesis imperfecta, osteoporosis-pseudoglioma syndrome, and high bone mass, but their role in predisposing to stress fracture is not clear [14-21]. Here we aimed to elucidate the possible role and significance of sequence variations in certain bone-related genes in the development of femoral neck stress fractures.

## Methods

### Subjects

Femoral neck stress fractures were studied because of the severity and potential consequences of these fractures [5,6]. All military conscripts who had suffered from femoral neck stress fractures and had been treated at the Finnish Defence Forces' military hospitals from 1970 to 1995 were invited to participate in a follow-up examination in 2002 or 2003. A total of 72 subjects were available for this follow-up. The diagnosis of stress fracture was originally based on accepted radiographic, scintigraphic, or MRI criteria [22,23]. The control group, collected also in the early 2000s, consisted of 120 Finnish conscripts who had not had stress fractures before or during military service, based on their military medical records and a questionnaire. The clinical characteristics of all the subjects are described in Table 1.

**Table 1:** Characteristics of cases and controls

Characteristics	Cases n = 72	Controls n = 120	p-value
Age	20.3 ± 1.6	18.9 ± 0.5	0.0005
Height	177.1 ± 6.0	179.6 ± 6.2	0.006
Weight	68.9 ± 9.6	77.3 ± 13.1	0.0005
BMI	22.0 ± 2.9	23.9 ± 3.5	0.0005
Smoking (N and % smokers)	29 (42.0%)	67 (56.8%)	0.069

Data are presented as mean ± SD

Finnish men become eligible for compulsory military service at the age of 18 years, and the duration of required service ranges from 6 to 12 months. Basic training lasts for 6 months and comprises a variety of exercises, ranging from marching, jogging, and cycling to drill and combat training, which involves heavy physical loading.

Information on the background variables was collected from the military medical records, including age, sex, height, weight, and smoking habit. The body mass index (BMI) of each conscript was calculated by dividing the body weight in kilograms by the square of the height in meters (kg/m^2^). Blood samples were collected from all subjects and controls. All the subjects were males between 18 and 27 years of age at the age of onset. The study was approved by the local ethics committee (Finnish Defence Forces, Helsinki, Finland), and signed informed consent was obtained from each subject.

### Scan for mutations in the coding regions of 5 candidate genes

Genomic DNA was isolated from the blood samples by standard procedures. Polymerase chain reaction amplification of 51 exons of *COL1A1*, 52 exons of *COL1A2*, and 23 exons of *LRP5 *was performed from 72 cases and 120 controls as previously described [14,20]. Primers were designed to amplify the 8 exons of *OPG *and the 5 exons of *ESR1 *(available on request). The polymerase chain reaction products were scanned for sequence variations by conformation-sensitive gel electrophoresis (CSGE) [14] and products that contained heteroduplexes were sequenced using an ABI PRISM 377 or 3100 Sequencer and the ABI PRISM BigDye Terminator Cycle Sequencing Ready Kit (Applied Biosystems, Foster City, CA).

### Genotyping of 15 SNPs in the VDR, CTR, IL-6, COL1A1, COL1A2, and LRP5 genes

The NCBI GenBank reference numbers and detection methods for each single nucleotide polymorphism (SNP) are shown in Table 2. Genotyping of the *VDR*, *CTR*, and *IL-6 *variations was performed as described previously [16,18,21,24,25]. Genes that have shown evidence of biological interactions were chosen as candidates for gene-gene interaction analysis [26,27], but only interaction combinations where sample size was large enough were evaluated.

**Table 2:** SNP allele frequencies between cases and controls

Gene	Sequence variation	Detection method	Allele	Allele frequencies (%)		p-value
					
				Controls	Cases	
*VDR*	c.2T > C, Met1Thr (rs10735810)	*Fok*I	C	153 (64)	90 (62)	
			T	87 (36)	54 (37)	NS
	c.1024+283G > A (rs1544410)	*Bsm*I	A	90 (38)	45 (31)	
			G	150 (63)	99 (69)	NS
	c.1056T > C, Ile352Ile (rs731236)	*Taq*I	C	88 (37)	45 (31)	
			T	152 (63)	99 (69)	NS
*IL6*	-174G > C (rs1800795)	*Nla*III	C	126 (52.5)	72 (50)	
			G	114 (47.5)	72 (50)	NS
*CTR*	c.1377C > T, Pro463Leu (rs1801197)	*Alu*I	C	73 (30)	35 (24)	
			T	167 (70)	109 (76)	NS
*COL1A1*	c.101+1024G > T (rs1800012)	Sequencing	G	204 (85)	123 (85)	
			T	36 (15)	21 (15)	NS
	c.1930-14T > C (rs2696247)	CSGE	C	41 (17)	28 (19)	
			T	199 (83)	116 (81)	NS
	c.3261C > T (rs2586488)	*Eco57*I	C	158 (66)	87 (60)	
			T	82 (34)	57 (40)	NS
*COL1A2*	c.280-68A > G (rs406226)	CSGE	A	206 (86)	120 (83)	
			G	34 (14)	24 (17)	NS
	c.1666-41G > A (rs2301643)	*Msl*I	A	32 (13)	24 (17)	
			G	208 (87)	120 (83)	NS
	c.2350-89ins38bp (rs3216902)	CSGE	insertion	151 (63)	96 (67)	
			no ins.	89 (37)	48 (33)	NS
*LRP5*	c.2007G > A, E644E (rs2277268)	Sequencing	G	231 (96)	131 (91)	
			A	9 (4)	13 (9)	**0.031**^1^
	c.2074G > A, V667M (rs4988321)	Sequencing	G	235 (98)	137 (95)	
			A	5 (2)	7 (5)	NS
	c.3432A > G, V1119V (rs556442)	Sequencing	A	200 (83)	112 (78)	
			G	40 (17)	32 (22)	NS
	c.4064C > T, A1330V (rs3736228)	Sequencing	C	227 (95)	134 (93)	
			T	13 (5)	10 (7)	NS

NS = not significant; GenBank Accession numbers *COL1A1 *NM_000088, *COL1A2 *NM_000089, *VDR *NM_000376, *LRP5 *NM_002335, *CTR *NM_001742; ^1^NS permutation p-value, p = 0.21.

### Statistical analysis

The potential deviation from the Hardy-Weinberg equilibrium was tested using the chi-square test. Disease association studies were performed on alleles and genotypes using the likelihood ratio chi-square test. Variables that differed between the cases and controls and were associated with the polymorphisms were included as covariables in multivariate analyses. Dominant, additive, recessive, and general genetic models were defined and tested for all of the polymorphisms. Haplotype frequencies and pair-wise linkage disequilibrium (D') and correlation coefficient (r^2^) values were established using Haploview software (MIT/Harvard Broad Institute Cambridge, MA) [28]. The haplotypes were reconstructed statistically from population genotype data using the PHASE program with the Markov chain method for haplotype assignment [29]. Potential risk or protective haplotypes were identified by comparing haplotype frequencies between the cases and controls using Fisher's exact probability test or the chi-square test. The robustness of the associations was evaluated with permutation tests (100 permutations).

Crude and adjusted odds ratios and their 95% confidence intervals (CIs) were calculated using the SPSS statistical package (SPSS, Chicago, IL), and interactions between the polymorphisms were investigated by stratification and logistic regression analysis. The statistical significance of a *p*-value was defined as the 5% level.

## Results

### Clinical findings

The basic characteristics of the cases and controls were compared and are shown on Table 1. The results indicated that the cases were smaller in size than the controls; i.e., they were shorter and had lower body weight and BMI. As the control group data were collected at a later time than those for the stress fracture patients, the height, weight, and BMI were also compared to those of healthy contemporaries of the cases. The findings were the same in that body weight and BMI were lower in cases than in their healthy contemporaries, indicating that the time discrepancy between controls and cases did not account for the differences in these parameters (data not shown). Unfortunately, DNA was not available for genetic analyses of the contemporary subjects. The risk of femoral neck stress fractures was significantly higher in subjects with low body weight and low BMI. The control group differed from the cases in regard to age, but no statistically significant association between age and BMI or BMI and smoking between control group and cases was detected (data not shown).

### Candidate gene analysis

Because sequence variations in *COL1A1*, *COL1A2*, *LRP5*, *OPG*, and *ESR1 *increase the risk of low bone mass or osteoporotic fractures, we first examined their possible role in the pathogenesis of stress fractures by scanning all their exons and exon boundaries in the 72 subjects and the 120 controls for mutations using CSGE [14,15,17,20]. The analysis revealed no putative disease-causing mutations. Several sequence variations were observed in these genes, but none of them was novel (as verified from the NCBI GenBank), and they were all detected in both stress fracture subjects and controls (data not shown).

### Association analyses of SNPs

To test for possible allelic associations, a total of 15 SNPs in 6 genes (*COL1A1*, *COL1A2*, *CTR*, *IL-6*, *VDR*, and *LRP5*) were genotyped. The genotype frequencies for 15 SNPs were all in Hardy-Weinberg equilibrium. A comparison of the resulting allele frequencies between the cases and controls (Table 2) suggested that the frequency of the *LRP5 *rs2277268 minor allele A was marginally elevated in the cases. The same allele was significantly associated with both low body weight (A: 68.6 ± 9.0 kg vs. G: 74.5 ± 12.7 kg, p = 0.036) and low BMI (A: 21.5 ± 2.5 kg/m^2 ^vs. G: 23.3 ± 3.4 kg/m^2^, p = 0.017). In addition, the *LRP5 *rs4988321 minor allele A was associated with lower height (A: 175.6 ± 4.6 cm vs. G: 178.8 ± 6.2 cm, p = 0.025), and the *VDR Bsm*I minor allele A was associated with higher BMI (A: 23.7 ± 3.7 kg/m^2 ^vs. G: 22.9 ± 3.2 kg/m^2^, p = 0.04). Because neither of the polymorphisms was associated with either age or smoking, only BMI was included as a covariate in the subsequent analyses.

The *COL1A1 *rs2586488 and *COL1A2 *rs3216902 SNPs were associated with stress fractures in a recessive model (Table 3), and the risk was increased in carriers of the *LRP5 *rs2277268 minor allele in comparison with non-carriers (OR = 2.72; 95% CI 1.10-6.73, p = 0.03). After adjusting for BMI, however, the observed associations lost their statistical significance. *VDR, CTR*, and *IL-6 *SNPs, did not significantly associate with stress fracture in any genetic model (Table 3).

**Table 3:** Genotype distributions, odds ratios (OR), and their 95% confidence intervals (CI) and p-values for the genetic models

						p-value			
						
Gene	SNPs ID	Genotype	Counts (Controls/Cases)	OR	95% CI	General association	Dominant	Additive	Recessive
*VDR*	rs10735810	C/C	41/29	1		0.24	0.57	0.80	0.18
		T/C	58/30	0.71	0.38-1.35				
		T/T	11/10	1.50	0.60-3.78				
	rs1544410	G/G	46/17	1		0.15	0.08	0.22	0.91
		G/A	58/25	0.54	0.28-1.01				
		A/A	16/10	0.78	0.32-1.91				
	rs731236	T/T	49/37	1		0.32	0.15	0.30	0.96
		T/C	54/25	0.61	0.32-1.16				
		C/C	17/10	0.78	0.32-1.90				
*IL6*	rs1800795	G/G	35/15	1		0.27	0.20	0.63	0.59
		G/C	56/42	1.75	0.85-3.61				
		C/C	29/15	1.21	0.51-2.88				
*CTR*	rs1922295	T/T	59/45	1		0.12	0.07	0.22	0.81
		T/C	49/19	**0.51**	**0.25-0.98**				
		C/C	12/8	0.87	0.33-2.32				
*COL1A1*	rs1800012	G/G	85/53	1		0.48	0.68	0.91	0.30
		G/T	34/17	0.80	0.41-1.58				
		T/T	1/2	3.21	0.28-36.25				
	rs2696247	T/T	189/100	1		0.45	**-**	**-**	-
		T/C	123/88	1.27	0.68-2.35				
	rs2586488	C/C	50/30	1		0.09	1.0	0.30	**0.04^1^**
		C/T	58/27	0.78	0.41-1.48				
		T/T	12/15	2.08	0.86-5.04				
*COL1A2*	rs406226	A/A	86/50	1		0.14	0.74	0.49	-
		A/G	34/20	1.01	0.53-1.94				
		G/G	0/2	-	-				
	rs2301643	G/G	88/50	1		0.13	0.56	0.35	-
		G/A	32/20	1.10	0.57-2.12				
		A/A	0/2	-	-				
	rs3216902	Ins/Ins	52/29	1		**0.05**	0.68	0.46	**0.03^2^**
		Ins/NoI	47/38	1.45	0.78-2.71				
		NoI/NoI	21/5	0.43	0.15-1.25				
*LRP5*	rs2277268	G/G	111/59	1		**0.03**	-	-	-
		G/A	9/13	**2.72**	**1.10-6.73**				
	rs4988321	G/G	115/65	1		0.13	-	-	-
		G/A	5/7	2.48	0.76-8.12				
	rs556442	A/A	82/43	1		0.36	0.23	0.17	0.30
		A/G	36/26	1.38	0.74-2.57				
		G/G	2/3	2.86	0.46-17.78				
	rs3736228	C/C	107/62	1		0.53	-	-	-
		C/T	13/100	1.33	0.55-3.21				

^1^OR = 2.37, 95% CI 1.04-5.40. ^2^OR = 0.35, 95% CI 0.13-0.98. Body mass index (BMI) was used as a covariate in the analyses.

### Linkage disequilibrium, haplotype and interaction analyses

**Figure 1:**
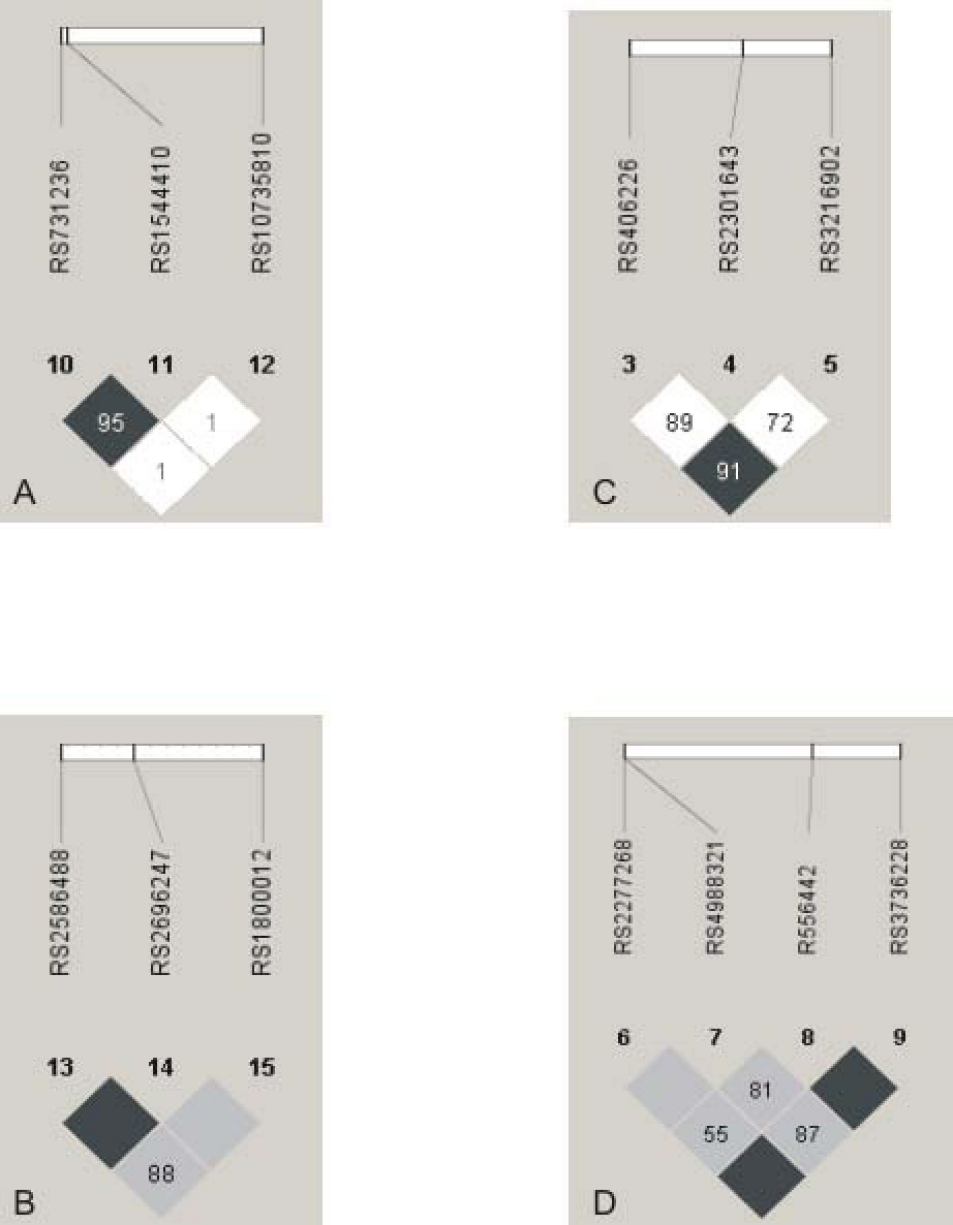
**A) Haploview linkage disequilibirum (LD) plot of the VDR SNPs, B) Haploview LD plot of the COL1A1 SNPs, C) Haploview LD plot of the COL1A2 SNPs, and D) Haploview LD plot of the LRP5 SNPs**. D' values are indicated in the figure.

The pairwise linkage disequilibrium between the SNPs within each gene was estimated in terms of D' and r^2^. A linkage disequilibrium plot for the *VDR, COL1A1*, *COL1A2*, and *LRP5 *SNPs is presented in Figures 1A-D. The SNPs within the *COL1A1 *and *COL1A2 *genes were in linkage disequilibrium (Figure 1). Of the eight *COL1A2 *haplotypes derived from the analysis, three were common in both cases and controls, but four rare haplotypes were detected only in controls. The *COL1A2 *haplotype frequencies were marginally different between the cases and controls (p = 0.0197, data not shown). The association of the phenotype with the *COL1A1 *haplotypes was not significant. In the case of the *VDR *gene, the *Fok*I (rs10735810) and the *Taq*I-*Bsm*I (rs731236 & rs1544410) SNPs were located in two different haplotype blocks. No association between the *VDR *haplotypes and stress fractures was detected.

Seven haplotypes were detected in the *LRP5 *gene. The A-G-G-C haplotype frequency was higher among cases than among controls (p = 0.031), and the risk of stress fractures was higher among its carriers than in non-carriers (OR 2.72; 95% CI 1.10 - 6.73). On the other hand, the A-G-G-C haplotype increased the risk of low BMI (OR 2.50; 95 % CI 1.03-6.07, p = 0.04), so the association between haplotype and stress fracture lost its statistical significance after adjusting for BMI (OR 2.04; 95% CI 0.79 - 5.22), suggesting that BMI mediates this association.

Interactions between the *CTR*-*VDR*, *VDR*-*LRP5 *and *CTR*-*VDR*-*LRP5 *genes were examined. Their contribution to the risk for stress fractures revealed an interaction between the *LRP5 *haplotype A-G-G-C and the *VDR *haplotype C-A (Table 4); i.e., the risk of femoral neck fracture was marginally increased in subjects without the C-A haplotype (OR 1.78; 95% CI 0.96 - 3.30), and increased 3.85-fold (95% CI: 1.16-12.84) in carriers of both the A-G-G-C and C-A haplotypes compared with carriers of only the C-A haplotype. The associations were attenuated after adjustment for BMI (OR 3.10; 95% CI 0.87-11.1 for a joint effect of haplotypes) suggesting, that BMI at least partly mediates the joint effect of the A-G-G-C and C-A haplotypes on stress fracture. The most significant finding of the present study was the observed interaction between a *CTR *minor allele C and the *VDR *C-A haplotype, the risk of fracture being significantly increased in subjects without the C allele or the C-A haplotype or without either one (OR = 3.22, 95% CI 1.38-7.49, p = 0.007) in comparison with carriers of both.

**Table 4:** *LRP5 *A-G-G-C haplotype and *VDR *C-A haplotype interaction

*LRP5*	*VDR*	Controls		Cases			
				
A-G-G-C	C-A	N	fr.	N	fr.	OR (95% CI)	p-value
- or +	-	50	0.383	37	0.444	1.78 (0.96-3.30)^1^	0.07
-	+	65	0.542	27	0.375	1.00	
+	+	5	0.042	8	0.111	**3.85 (1.16-12.84)^2^**	**0.028**

^1^1.85 (0.96-3.55) after adjustment for body mass index (BMI)

^2^3.10 (0.87-11.1) after adjustment for body mass index (BMI)

## Discussion

The significance of physical and mechanical risk factors in the development of stress fractures is well established [9]. The present findings indicate that genetic factors may also play a role in the development of femoral neck stress fractures. We found an interaction between the *CTR *C allele and the *VDR *C-A haplotype, and the risk of stress fractures was 3-fold higher in military conscripts lacking either one or both compared to carriers of both. In addition, the *LRP5 *gene haplotype A-G-G-C conferred almost a 3-fold increased risk for developing femoral neck stress fractures, and a 4-fold increased risk in combination with the *VDR *C-A haplotype, although these associations were mediated by low body weight and BMI and require further investigation.

Because of the important role of CTR in bone homeostasis, polymorphisms of this gene have been studied with regard to common bone parameters and disorders. Several studies report an association between the *CTR *polymorphism *Alu*I and lumbar spine and femoral neck BMD in both men and women, but the reports have been somewhat contradictory. Studies in postmenopausal women revealed that the CC genotype is more common in non-osteoporotic women than the TT genotype [30,31], and that the TT genotype is associated with lower lumbar spine and femoral neck BMD and increases the predisposition for osteoporosis [32,33]. The opposite finding was suggested by Braga et al. [34] who reported that the CC genotype is associated with decreased BMD and is more common in men with hip or vertebral fractures than in control subjects [34]. In our study, the *CTR *allele C together with a *VDR *C-A haplotype appeared to protect subjects from fractures.

Polymorphisms in *CTR *and *VDR *are associated with BMD in Spanish women [35]. The observed interaction between a *CTR *minor allele and the *VDR *C-A haplotype and their association with stress fractures may be explained by the inhibitory effect of these proteins on parathyroid hormone production. CTR and VDR are both involved in sustaining normocalcemia by inhibiting the production of parathyroid hormone [36]. The observed allele-haplotype interaction may have an effect on the regulatory role of the proteins and therefore on control of Ca levels.

VDR also has independent effects on bone biology and may play a role in bone pathologies such as stress fractures. VDR is essential for 1,25(OH)_2_D_3 _to induce the calcemic and phosphatemic effects that normally result in bone mineralization and remodelling [37]. *VDR *genotypes increase the risk for low BMD and osteoporotic fractures [16,38] and *VDR *knock-out mice develop a low bone mass phenotype with hypocalcemia, hypophosphatemia, and elevated 1,25(OH)_2_D_3 _levels [39]. In addition, reduced serum 25(OH)D levels might predispose young men to stress fractures [40].

The present study indicates that the association of the *LRP5 *haplotype and *LRP5*-*VDR *interaction with stress fractures is mediated by low body weight and BMI, but more research is needed before any definitive conclusions can be drawn from these findings. The function of LRP5 in bone development, however, is indisputable [41]; mutations in *LRP5 *cause various bone disorders [20,42] and polymorphisms are associated with BMD and bone mineral content in general [43], but also with reduced BMD and fractures [44]. Mouse studies demonstrated that mutations in *Lrp5 *affect bone formation sensitivity in response to normal mechanical loading [45,46], and thus the *LRP5 *haplotype A-G-G-C might affect bone sensitivity and response to mechanical loading. It is possible that bone in the lighter-weight conscripts is initially adjusted to lower load bearing and when mechanical loading sharply increases in the military service, the genetically set response might not adjust rapidly enough to react to the increased loading, thus putting lighter weight conscripts at higher risk for stress fractures. Our results support earlier findings that low weight (before and/or during military service) increases the risk of stress fractures [3,47]. Body weight is an important predictor of BMD [48], and BMI and obesity have been shown in a family-based analysis to be associated with *LRP5 *polymorphisms [49], underlining the role of *LRP5 *also in weight regulation.

One limitation of the present study is the discrepancy in the collection periods of case and control groups. The most important physical variable was weight, because reduced weight and BMI mediate the association of the *LRP5 *haplotype and the *LRP5-VDR *interaction with femoral neck stress fractures. Interestingly, however, the same difference in weight and BMI was also observed between the cases and their healthy contemporaries, suggesting that the difference was not time-dependent. This verification is important because the mean BMI and the number of overweight conscripts has consistently increased towards the end of 20^th ^century, whereas the physical fitness of conscripts has declined [50]. Unfortunately, other extrinsic factors that could have changed during the 30 years scale (e.g. in nutrition or exercise) have not been examined because of the limited amount of information available. The results of the study should be interpreted with moderation and replication of the study is needed to confirm the present findings. For future studies, larger sample sizes are desirable to gain more statistical power in the analyses. In addition, functional studies on the present genetic findings are needed to elucidate the relevance of these genetic associations to femoral neck stress fractures.

## Conclusions

Our findings suggest that genetic factors may play a role in the development of stress fractures in individuals subjected to heavy exercise and mechanical loading. The present results can be applied to the design of future studies that will further elucidate the genetics of stress fractures.

## Authors' contributions

JK and HH carried out molecular genetic studies and drafted the manuscript. SS and SB performed the statistical analyses. HP, JPR, TS, JO, LAK and MM conceived of the study, participated in its design and coordination and helped to draft the manuscript. All authors read and approved the manuscript.
